# Hepatocyte growth factor-mediated apoptosis mechanisms of cytotoxic CD8^+^ T cells in normal and cirrhotic livers

**DOI:** 10.1038/s41420-023-01313-4

**Published:** 2023-01-19

**Authors:** Quanyu Chen, Min Yan, Heng Lin, Jiejuan Lai, Zhiqing Yang, Deyu Hu, Yuanyu Deng, Saiyu Shi, Ling Shuai, Leida Zhang, Hongyu Zhang, Lianhua Bai

**Affiliations:** 1 Hepatobiliary Institute, Southwest Hospital, Army Medical University, No. 30 Gaotanyan, ShapingBa Distract, 400038 Chongqing, China; 2 Key Laboratory of Freshwater Fish Reproduction and Development, Ministry of Education, Laboratory of Molecular Developmental Biology, School of Life Sciences, Southwest University, 400715 Beibei, Chongqing China; 3 Department of Special Medicine, Shanxi Medical University, 030000 Taiyuan, China; 4 Bioengineering College, Chongqing University, No. 175 Gaotan, ShapingBa Distract, 400044 Chongqing, China; 5 School of Basic Medicine, Army Medical University, No. 30 Gaotanyan, ShapingBa Distract, 400038 Chongqing, China; 6 Present Address: Hepatobiliary Institute, Southwest Hospital, Army Medical University, No. 30 Gaotanyan, ShapingBa Distract, 400038 Chongqing, China

**Keywords:** Apoptosis, Growth factor signalling

## Abstract

Intrahepatic stem/progenitor cells and cytotoxic CD8^+^ T cells (CD8^+^ T cells) in the cirrhotic liver undergo apoptosis, which potentially facilitates progression to cancer. Here, we report that hepatocyte growth factor (HGF) signaling plays an important role in promoting normal and damaged liver CD8^+^ T cell Fas-mediated apoptosis through its only receptor, c-Met. In addition to binding with HGF, c-Met also binds to Fas to form a complex. Using a diethylnitrosamine (DEN)-induced liver fibrosis/cirrhosis mouse model, immunostaining, and terminal deoxynucleotidyl transferase (TdT) dUTP nick-end labeling (TUNEL) staining, we found that HGF secretion was significantly higher at 10 weeks post-DEN, the liver cirrhotic phase (LCP), than at 3 weeks post-DEN, the liver fibrotic phase (LFP). Correspondingly, differences in CD8^+^ T cell proliferation and apoptosis were noted between the two phases. Interestingly, staining and TUNEL assays revealed lower smooth muscle actin (α-SMA)^+^ cell apoptosis, a marker for hepatic stellate cells (HSCs), in the LFP group than in the LCP group, which suggested a beneficial correlation among HGF, CD8^+^ T cells and HSCs in improving the fibrotic load during damaged liver repair. In cultures, when met different concentrations of recombinant HGF (rHGF), phytohemagglutinin (PHA)-stimulated naive mouse splenic CD8^+^ T cells (pn-msCD8^+^ T cells) responded differently; as increases in rHGF increased were associated with decreases in the clonal numbers of pn-msCD8^+^ T cells, and when the rHGF dose was greater than 200 ng/mL, the clonal numbers significantly decreased. In the presence of 400 ng/mL rHGF, the death-inducing signaling complex (DISC) can be directly activated in both nsCD8^+^ T cells and healthy human peripheral blood CD8^+^ T cells (hp-CD8^+^ T cells), as indicated by recruitment of FADD and caspase-8 because DISC forms via the recruitment of FADD and caspase-8, among others. These findings suggest that Fas-mediated apoptosis, may also indicate a regulatory role of HGF signaling in hepatic homeostasis.

## Introduction

Hepatic niche apoptosis is a common mechanism of liver injury and contributes to the development and progression of liver fibrosis and cirrhosis [1, 2]. Importantly, hepatocytes [3, 4], hepatic stem/progenitor cells (HSPs) [5] and immune CD8^+^ T cells [6] contribute to liver homeostasis (LH) [7, 8] and the progression of liver fibrosis and cirrhosis. Chronic liver diseases, such as hepatitis B virus (HBV) and nonalcoholic fatty liver disease (NAFLD), promote liver inflammation and injury, which can proceed to cirrhosis-mediated end-stage liver disease (ESLD) and require liver transplantation [9]. Aberrant Fas (a death receptor) activation is an important step in apoptosis [10]. Human and animal studies have shown that Fas expression is upregulated in fibrotic and cirrhotic livers compared with normal controls. Other studies have established a direct connection between Fas and the increased sensitivity to liver damage observed in fibrotic/cirrhotic livers [11–13]. c-Met (hepatocyte growth factor [HGF] only receptor) [14] plays an important role in protecting hepatic cells against apoptosis through either an intracellular axis involving PI3K-Akt activation or an extracellular pathway involving sequestration of the death receptor Fas [15–18]. In a previous study using a diethylnitrosamine (DEN)-induced liver fibrotic/cirrhotic animal model, we demonstrated that endogenous HSPs survival is better in the liver fibrotic phase (LFP, 3–6 weeks post-DEN) and that death via apoptosis occurs in the liver cirrhotic phase (LCP, 7–10 weeks post-DEN) [5]. In the present study, we found similar evidence that liver tissue resident CD8^+^ T cells in diseased liver tissue exhibited the similar fate. These cells survive better in the LFP to promote fibrosis resolution, but death via apoptosis occurs in the LCP. However, all of the underlying mechanisms remain largely unknown. Known higher HGF secretion in human fatty livers than in normal livers [19] and its signaling system as cytotoxic in inducing apoptosis on some cell settings [20, 21], we hypothesize that HGF may influence the survival of CD8^+^ T cells in both naive and diseased livers. We also hypothesize that excess amounts of HGF may induce dissociation of c-Met from Fas in CD8^+^ T cells and promote Fas accumulation to enhance sensitivity to its ligand, FasL, which leads to Fas-mediated apoptosis [22]. Thus, interventions could be developed to perturb this mechanism.

## Results

### Higher HGF secretion promotes lcpCD8^+^ T cell apoptosis in a mouse model of liver fibrosis/cirrhosis induced by DEN

**Fig. 1:**
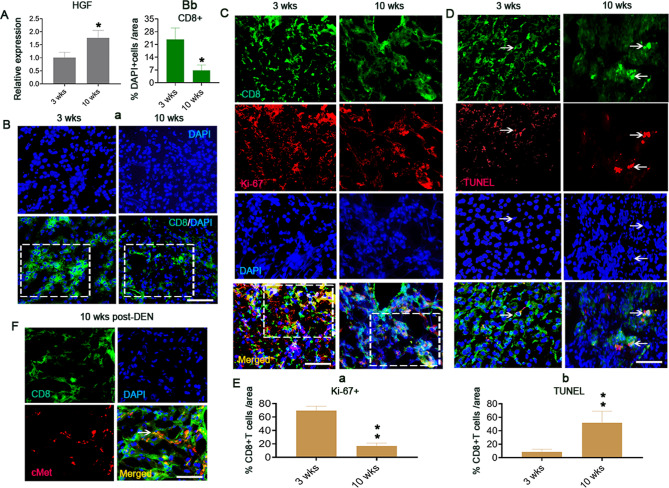
Higher HGF secretion induces CD8^+^ T cell apoptosis in the liver cirrhotic period (LCP) of DEN-induced liver fibrosis/cirrhosis mouse model. **A** qRT‒PCR assay of HGF expression in the liver tissues at 3 and 10 weeks post-DEN, *n* = 3. **B**(a, b) Immunofluorescence staining for the percentage of CD8^+^ T cells with liver tissue-resident (DAPI^+^) cells at 3 and 10 weeks post-DEN (a, green, boxes) and quantification (b). Scale bar = 200 µM, *n* = 6. **C**, **D** Double immunofluorescence staining of Ki-67 (red, **C**) and TUNEL (red, **D**) within CD8^+^ T cells (green) in the diseased liver tissues at 3 and 10 weeks post-DEN (brown color after merging in boxes, **C**, **D**). Scale bar = 200 µM, *n* = 6/staining marker. **E**(a, b) Quantification of the counts in **C** and **D**. **F** Double immunofluorescence staining of c-Met (red) and CD8 (green) in liver tissues at 10 weeks post-DEN. The arrow marks c-Met-expressing CD8^+^ T cells (brown). Scale bar = 200 µM, *n* = 6. At least three independent experiments were performed, and the data are presented as the means ± SDs; *p < 0.05 and *p < 0.001 vs. 10 weeks post-DEN (Student’s *t* test).

Higher HGF expression has been observed in the diseased liver than in the normal liver [19]. By quantitative real-time PCR (qRT‒PCR), we found that HGF gene expression at 10 weeks post-DEN (in the LCP) [5] was approximately twofold higher than that at 3 weeks post-DEN (in the LFP) [5] (Fig. 1A). These results suggest the presence of increase in the HGF levels in the cirrhotic niche during disease development. Correspondingly, by immunofluorescence staining, we found higher numbers of CD8^+^ T cells (green) at 3 weeks post-DEN than at 10 weeks post-DEN (Fig. 1Ba, boxes, Fig. 1Bb). Thus, the HGF levels in the LCP potentially affect CD8^+^ T cell activity. Through double staining for Ki-67 (red, a proliferation marker) and TUNEL (red, an apoptosis marker), we revealed decreased numbers of Ki-67^+^ cells within CD8^+^ T cells (green) at 10 weeks compared with those at 3 weeks (Fig. 1C, boxes, brown color after merging, Fig. 1Ea), and Ki-67^+^/CD8^+^ T cells exhibited lower TUNEL expression (red) at 10 versus 3 weeks post-DEN (Fig. 1D, arrows, brown color after merging, Fig. 1Eb). These results suggest that the lower levels of CD8^+^ T cells in the high-HGF-expression cirrhotic liver niche (lcpCD8^+^ T cells) can be attributed to death via apoptosis. Moreover, by double immunofluorescence staining, we confirmed that the lcpCD8^+^ T cells (green, Fig. 1F) at 10 weeks post-DEN expressed the HGF receptor c-Met (red, Fig. 1F, brown color after merging, denoted by an arrow), which suggests that HGF in cirrhotic niche affects the lcpCD8^+^ T cell survival through the c-Met receptor. These observations suggest that the vulnerability of CD8^+^ T cells to apoptotic death in the liver cirrhotic period is associated with higher HGF levels.

### Higher HGF levels directly induce apoptosis of naive mouse splenic CD8^+^ T cells (n-msCD8^+^ T cells)

**Fig. 2:**
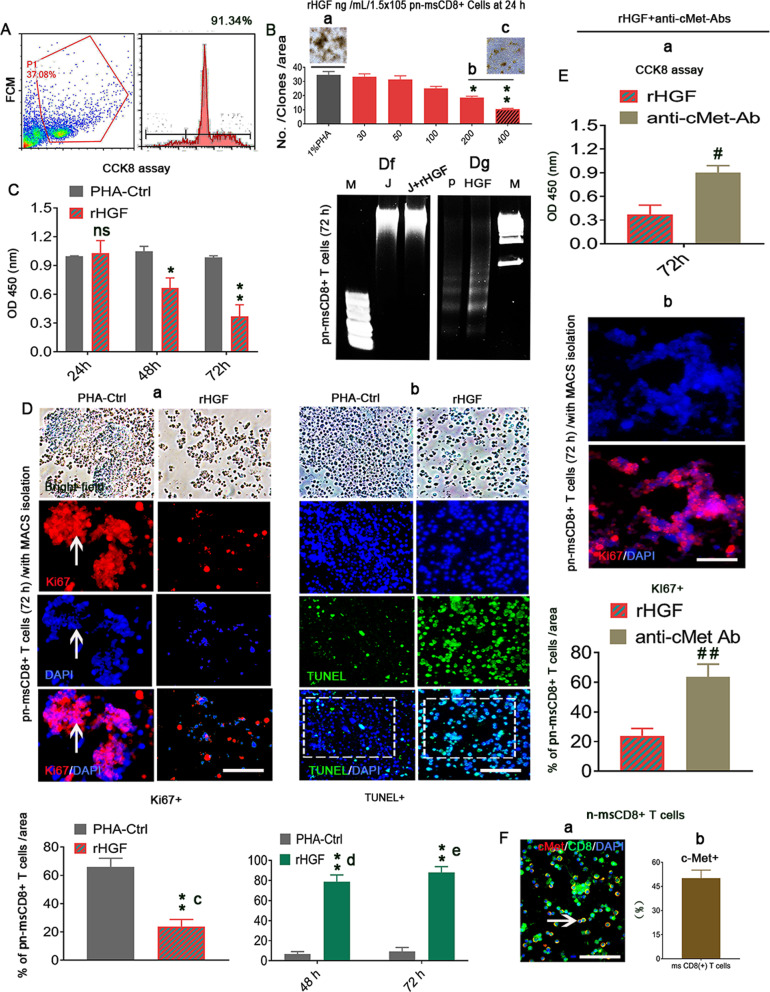
rHGF (400 ng/mL) inhibits msCD8^+^ T cell proliferation and promotes apoptosis that mediated by c-Met receptor. **A** Analysis of the purity of n-msCD8^+^ T cells isolated using the MACS method with CD8α antibody as assessed by FCM (*n* = 3). **B** pn-msCD8^+^ T cells (1 × 10^5^) were cultured and treated with different doses of rHGF for 24 h (*n* = 3). **C** CCK-8 assays were performed for the analysis of pn-msCD8^+^ T cell growth between the rHGF-treated and nontreated groups at 24, 48, and 72 h after rHGF administration (*n* = 3/time point). **D**(a–g) Immunofluorescence staining for Ki-67^+^ cells among MACS-purified pn-msCD8^+^ T cells within DAPI^+^ cells in the present and absence of rHGF treatment for 72 h (a, c, arrows denote proliferation T cell clones (red), *n* = 6; TUNEL (green) assay of cell apoptosis after rHGF treatment for 48 (d) and 72 h (b, green, e), scale bar = 200 µM, *n* = 6; 72-h rHGF (400 ng/mL)-treated pn-msCD8^+^ T cells (g, *n* = 3) and Jurkat T cells (J) (f, *n* = 3) were also subjected to gel electrophoresis to detect DNA fragments. **E**(a, b) Inhibitory effects of an anti-c-Met-Ab on pn-msCD8^+^ T cell proliferation as assessed by CCK-8 assay (a, *n* = 3) and immunostaining (b, red, boxes, *n* = 3) 24 h before 400 ng/mL rHGF administration, scale bar = 200 µM. **F** Immunofluorescence staining for c-Met (red) in pn-msCD8^+^ T cells (green, *n* = 6, an arrow denotes c-Met-expressing n-msCD8^+^ T cells, brown color after merging) and quantification of c-Met expression by FCM (brown bars, *n* = 3). Scale bar = 200 µm. At least three independent experiments were performed, and the data are presented as the means ± SDs. The asterisks indicate a statistically significant difference; *p < 0.05 and **p < 0.001 vs. PHA-Ctrls; ^#^*p* < 0.05 and ^##^*p* < 0.001 vs. rHGF; ns represents no significance (Student’s *t* test).

Because the lcpCD8^+^ T cell apoptosis in the liver cirrhotic period is correlated with higher levels of HGF, we assessed whether higher HGF levels could also impact the n-msCD8^+^ T cells. Here, C57BL/6 inbred n-msCD8^+^ T cells were isolated using magnetic-activated cell sorting (MACS) beads and cocultured with different concentrations of recombinant hepatocyte growth factor (rHGF) at different time points. Flow cytometry (FCM) revealed that the purity of n-msCD8^+^ T cells was approximately 91.34% (Fig. 2A). The treatment of phytohemagglutinin (PHA)-stimulated n-msCD8^+^ T cells (pn-msCD8^+^ T cells) with different concentrations (from 30 to 400 ng/mL) of rHGF for 24 h, and up to 200 ng/mL rHGF induced a significant (*p < 0.05) decrease in the number of clones (Fig. 2Bb) compared with those obtained with PHA-stimulated n-msCD8^+^ T cell controls (1%PHA, Fig. 2Ba), and an even more significant (**p < 0.01) reduction was observed with 400 ng/mL rHGF (Fig. 2Bc). Thus, 400 ng/mL rHGF was selected as the experimental concentration for use throughout the study. Using CCK8 assays, we found a significant decrease in pn-msCD8^+^ T cell growth after 48 h and particularly at 72 h (**p < 0.01, Fig. 2C). Immunostaining further revealed that coculture with 400 ng/mL rHGF markedly decreased the numbers of Ki-67^+^/pn-msCD8^+^ T cells at 72 h compared with those of PHA-Ctrl cells (Fig. 2Da, arrows, Fig. 2Dc). In addition, the corresponding levels of TUNEL-positive cells were increased at both 48 (Fig. 2Dd) and 72 h (Fig. 2Db, boxes, Fig. 2De). To establish whether apoptosis is the mode of cell death observed among HGF-treated pn-msCD8^+^ T cells, DNA fragmentation was analyzed. The levels of fragmentation in HGF-treated pn-msCD8^+^ T cells were compared with those in Jurkat T cells (J), which do not express c-Met [19]. DNA fragments from n-msCD8^+^ T cells treated with 400 ng/mL rHGF for 72 h were subjected to agarose gel electrophoresis. A typical pattern of a DNA ladder in multiples of approximately 200 base pairs was observed (Fig. 2Dg), which suggested internucleosomal DNA fragmentation. In contrast, this pattern was not observed with DNA from PHA-Ctrl, J alone and J treated with rHGF (Fig. 2Df, p indicates PHA, J indicates Jurkat T cells). In addition, the ablation of rHGF signaling using anti-c-Met monoclonal antibodies (anti-c-Met-Ab, 50 ng/mL) 24 h before rHGF treatment and the CCK8 assay revealed negation of the effects on cell growth (Fig. 2Ea) and proliferation (Ki-67^+^, Fig. 2Eb) at 72 h compared with those observed with rHGF treatment alone. Additionally, double immunofluorescence staining (Fig. 2Fa) revealed approximately 50% c-Met protein expression (red) on n-msCD8^+^ T cells (Fig. 2Fb, an arrow denotes merged staining of CD8^+^ T cells, brown), which suggests that HGF triggers pn-msCD8^+^ T cell apoptosis through c-Met. These observations indicate that higher HGF levels could directly trigger apoptosis of n-msCD8^+^ T cells.

### rHGF (400 ng/mL) enhances death-inducing signal complex (DISC) formation in n-msCD8^+^ T cells

**Fig. 3:**
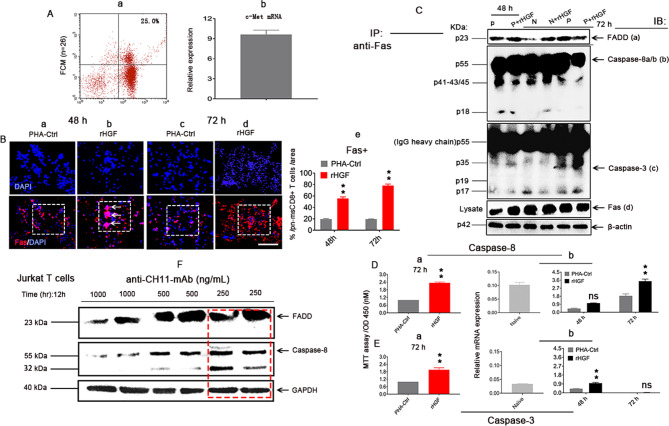
rHGF (400 ng/mL) enhances DISC formation to promote Fas-mediated apoptosis in both naive and PHA-stimulated msCD8^+^ T cells. **A**(a, b) c-Met expression in n-msCD8^+^ T cells at the protein (a, *n* = 3) and mRNA (b, *n* = 3) levels identified by FCM and qRT‒PCR assays, respectively. **B**(a–e) Immunofluorescence staining for Fas expression (red) in MACS-purified pn-msCD8^+^ T cells (denoted in blue) after 48 (a, b, boxes, arrows denote Fas aggregation) and 72 h (c, d, boxes) of 400 ng/mL rHGF treatment and quantification (e) compared with the PHA-control groups. Scale bar = 200 µM, *n* = 6/time point; **C**(a–d) IP with a Fas antibody followed by IB for FADD (a, *n* = 2), caspase-8 (b, *n* = 2), and caspase-3 (c, *n* = 2) of cell lysates only for Fas control protein (d, *n* = 2) after 48 h of 400 ng/mL treatment. **D**(a, b) MTT assay (a) and RT-qPCR (b) were used for the assessment of caspase-8 expression in the presence or absence of 400 ng/mL rHGF for 48 (b) and 72 h (a, b), *n* = 10. **E**(a, b) MTT assay (a) and RTq-PCR (b) were performed for the assessment of caspase-3 expression in the presence or absence of 400 ng/mL rHGF for 48 (b) and 72 h (a, b), *n* = 10. **F** Anti-CH11-mAb (250 ng/mL, red box) was selected for the treatment of Jurkat T cells (J) based on the experimental results to determine the ideal concentration, *n* = 2. At least three independent experiments were performed, and the data are presented as the means ± SDs; *p < 0.05 and **p < 0.001 vs. PHA-Ctrls; ns represents no significance (Student’s *t* test).

Because n-msCD8^+^ T cells express c-Met at both the protein (Figs. 2F and 3Aa) and mRNA levels (Fig. 3Ab), we subsequently investigated the mechanisms involved in the induction of apoptosis in these cells. We tested the hypothesis that a high concentration of rHGF induces the apoptosis pathway through the death receptor Fas. To do this, pn-msCD8^+^ T cells were cocultured with 400 ng/mL rHGF for 48 and 72 h. We found that Fas (red) was more highly expressed in these MACS-purified CD8^+^ T cells (blue, Fig. 3Bb, d) than in controls (PHA-Ctrl, Fig. 3Ba, c, boxes, Fig. 3e), and oligomerization was noted, particularly at 48 h (Fig. 3Bb, arrows in a box). To explore the mechanism of HGF and Fas communication, the status of the apoptotic mediators was examined starting with the most proximal events in the Fas apoptosis pathway on n-msCD8^+^ T cells, the hallmark of DISC formation. Upon oligomerization, Fas rapidly recruits Fas-associated death domain (FADD) and caspase-8 to the cytoplasmic death domain of Fas. As shown in Fig. 3Ca–d, the treatment of both n- (N) and pn-msCD8^+^ T cells with 400 ng/mL rHGF resulted in rapid formation of DISC. Recruitment of FADD (a) and caspase-8a/b (b) to Fas was detectable within 48 and 72 h of rHGF addition, as identified by immunoprecipitation (IP, to Fas) followed by immunoblotting (IB) using anti-FADD and anti-caspase-8/3 antibodies. A similar trend was found for caspase-8 in these cells by the 3-(4,5-dimethylthiazol-2-yl)-2,5-diphenyltetrazolium bromide (MTT) assay (Fig. 3Da) and quantitative real-time polymerase chain reaction (RT‒qPCR) (Fig. 3Db) at 72 h. Caspase-8 is the initiator caspase and is known to directly activate caspase-3, one of the effector caspases in the apoptosis pathway [23], so, the treatment of the same cells with 400 ng/mL rHGF also activated caspase-3 at both 48 (Fig. 3Eb) and 72 h (Fig. 3Ea; Cc), and the dissociation of c-Met from Fas caused by rHGF was not due to a decrease in the level of Fas protein after these treatments because a-c analysis of these same cell lysates indicated no changes in the overall protein levels of Fas (Fig. 3Cd). Because J express Fas [24], the CH-11 (anti-Fas/Apo-1) [25] monoclonal antibody (anti-CH11-mAb) also elicited DISC formation (Fig. 3F, red box is the selected concentration throughout the study). These observations indicate that DISC formation can be stimulated by high HGF levels and that Fas and rHGF cooperate to induce n-msCD8^+^ T cell apoptosis.

### c-Met-Fas dissociation in n-msCD8^+^ T cells treated with 400 ng/mL rHGF

**Fig. 4:**
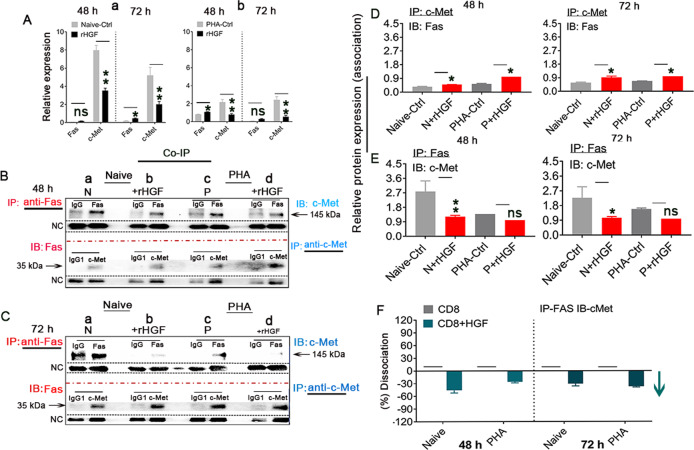
rHGF regulates the c-Met/Fas molecular mode in naive and PHA-stimulated msCD8^+^ T cells. **A** qRT‒PCR analysis of Fas and c-Met expression in naive (a) and PHA-stimulated (b) msCD8^+^ T cells 48 and 72 h after 400 ng/mL rHGF administration, *n* = 6. **B**, **C** A Co-IP assay was performed by IP with Fas or c-Met antibody followed by IB using an affinity-purified anti-c-Met-Ab or anti-Fas-Ab to evaluate the association between c-Met and Fas on naive (a, b, *n* = 3) and PHA-stimulated (c, d, *n* = 3) msCD8^+^ T cells in the presence (b, d) or absence (a, c) of 400 ng/mL rHGF treatment for 48 h (**B**(a–d)) and 72 h (**C**(a-d), *n* = 3/naive and PHA groups). **D** Quantification of the association between c-Met and Fas by IP with c-Met antibody followed by IB for Fas performed at 48 and 72 h (*n* = 6). **E** IP with Fas antibody followed by IB for c-Met performed at 48 and 72 h (*n* = 6). **F** Analysis of the dissociation rate of c-Met from Fas after 48 and 72 h of treatment with 400 ng/mL rHGF (the values of the samples marked with short lines were precisely 0). The data are presented as the means ± SDs of at least three independent experiments; original magnification = ×400; scale bar = 200 µm. The asterisks indicate a statistically significant difference; *p < 0.05 and **p < 0.001 vs. Naive-Ctrl and PHA-Ctrls; ns represents no significance (Student’s *t* test).

Because DISC formation along with increased Fas expression and decreased c-Met expression was revealed by RT‒qPCR assay in both naive (n-msCD8^+^ T cells) (Fig. 4Aa) as well as pn-msCD8^+^ T cells (Fig. 4Ab) mouse splenic CD8^+^ T cells undergoing Fas-mediated apoptosis 48 and 72 h after treatment with 400 ng/mL rHGF, we hypothesized that crosstalk may occur between HGF signaling (c-Met) and the death receptor Fas. To test this hypothesis, we cultured naive (N) and PHA (P)-stimulated n-msCD8^+^ T cells with 400 ng/mL rHGF for 48 (Fig. 4B) and 72 h (Fig. 4C) and then examined the association by a coimmunoprecipitation (IP) assay. msCD8^+^ T cell lysates from both naive and PHA-stimulated cultures were immunoprecipitated with antibodies against either Fas (anti-Fas, red) or c-Met (anti-c-Met, blue) and then subjected to immunoblotting (IB) with anti-c-Met (blue) or anti-Fas (red) antibodies. In non-rHGF-treated cells (time zero, N, P, Fig. 4Ba, c, Ca, c), c-Met immunoprecipitated with Fas. This association can be abrogated by treatment with 400 ng/mL rHGF for 48 and 72 h (Fig. 4Bb, d, Cb, d), and the dissociation caused by rHGF was not due to a decrease in the level of c-Met or Fas protein following these treatments because an analysis of these same cell lysates indicated no changes in the overall protein levels of c-Met and Fas (Fig. 4B, C, denoted NC). Further analysis by either IP c-Met IB Fas (Fig. 4D) or IP Fas and IB c-Met (Fig. 4E) revealed that the association between c-Met and Fas decreased from 48 to 72 h, leading to increased levels of free Fas (Fig. 4Da, b) and decreased levels of c-Met (Fig. 4Ea, b). An assessment of the dissociation caused by rHGF revealed that c-Met dissociation was also decreased in these cells (Fig. 4F, an green arrow) compared with controls (cells only, Fig. 4F, denoted by short horizontal lines), which suggested the disassociation of c-Met with Fas in n-smCD8^+^ T cells with increase in the post-rHGF treatment time . These data indicate that c-Met and Fas pre-exist as a complex in naive msCD8^+^ T cells and that high HGF concentrations disrupt their interaction with one another. This condition results in the aggregation of Fas (Fig. 3B, arrows), which causes higher sensitivity to FasL and thus leads to apoptosis, and whether this phenomenon could have physiological significance, such as maintaining hepatic homeostasis, should be further investigated.

### Liver tissue-resident CD8^+^ T cell apoptosis in the liver cirrhotic period affects fibrosis resolution in DEN-induced liver fibrosis/cirrhosis mouse model

**Fig. 5:**
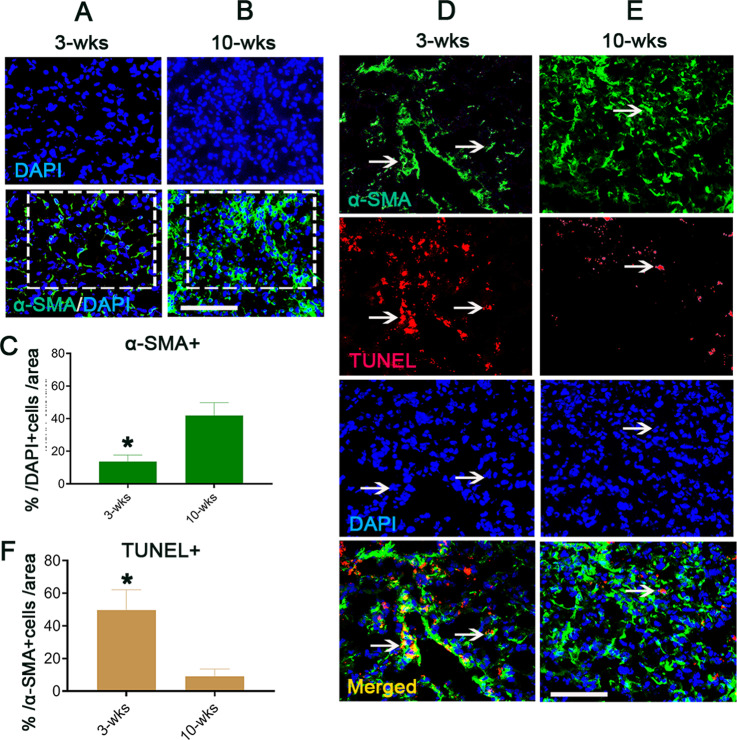
Liver resident CD8^+^ T cell apoptosis in the LCP period seems to promote fibrosis via protecting HSCs from apoptosis. **A**–**C** Immunofluorescence staining for fibrotic/cirrhotic liver tissue-resident α-SMA^+^ HSCs (green) at 3 (**A**, a box) and 10 weeks (**B**, a box) post-DEN and quantification (**C**). Scale bar = 200 µM, *n* = 6. **D**–**F** TUNEL staining (red) to target α-SMA^+^ HSCs labeled by red fluorescence (red) at 3 (**D**, arrows) and 10 (**E**, arrows) weeks post-DEN and quantification of the numbers of double-stained TUNEL and α-SMA^+^ cells (**F**). Scale bar = 200 µM, *n* = 6/each staining. At least three independent experiments were performed, and the data are presented as the means ± SDs; *p < 0.05 vs. 10 weeks post DEN (Student’s *t* test).

CD8^+^ tissue-resident T cells promote liver fibrosis resolution in other animal models of liver fibrosis disease by inducing apoptosis of hepatic stellate cells (HSCs) [26]. To address whether the different behaviors of CD8^+^ T cells in fibrotic (LFP) and cirrhotic (LCP) livers alter the effect of the hepatic fibrosis load in our fibrosis/cirrhosis DEN model, we performed immunofluorescence staining of alpha smooth muscle actin (α-SMA)-positive cells, a marker of HSC activation [27], in liver tissues 3 (in the LFP) and 10 weeks (in the LCP) [5] post-DEN. Significantly higher numbers of α-SMA^+^ cells (green) were observed at 10 weeks (Fig. 5B) compared with 3 weeks (Fig. 5A, boxes, Fig. 5C). Through double immunofluorescence staining for α-SMA (green, Fig. 5D, E, top panels) and a TUNEL assay (red, Fig. 5D, E, second panels from top) for examining HSC apoptosis (grown color after merging, bottom panels, arrows), we further found higher numbers of α-SMA^+^ cells costained with TUNEL at 10 weeks (Fig. 5E) than at 3 weeks (Fig. 5D, F). This result suggests that during the liver fibrotic period, CD8^+^ T cells are indeed beneficial for fibrosis resolution by triggering HSC apoptosis [26]. As the disease progresses into the cirrhotic period, the niche CD8^+^ T cells undergoing apoptosis may induce loss of inhibition mediated by these cells [26]. These observations indicate that CD8^+^ T cell activity in the damaged (fibrosis/cirrhosis) liver niche is associated with the survival fate of HSCs during disease development.

### c-Met-Fas dissociation in CD8^+^ T cells from healthy human peripheral blood (hp-CD8^+^ T cells) can also be induced by 400 ng/mL rHGF

**Fig. 6:**
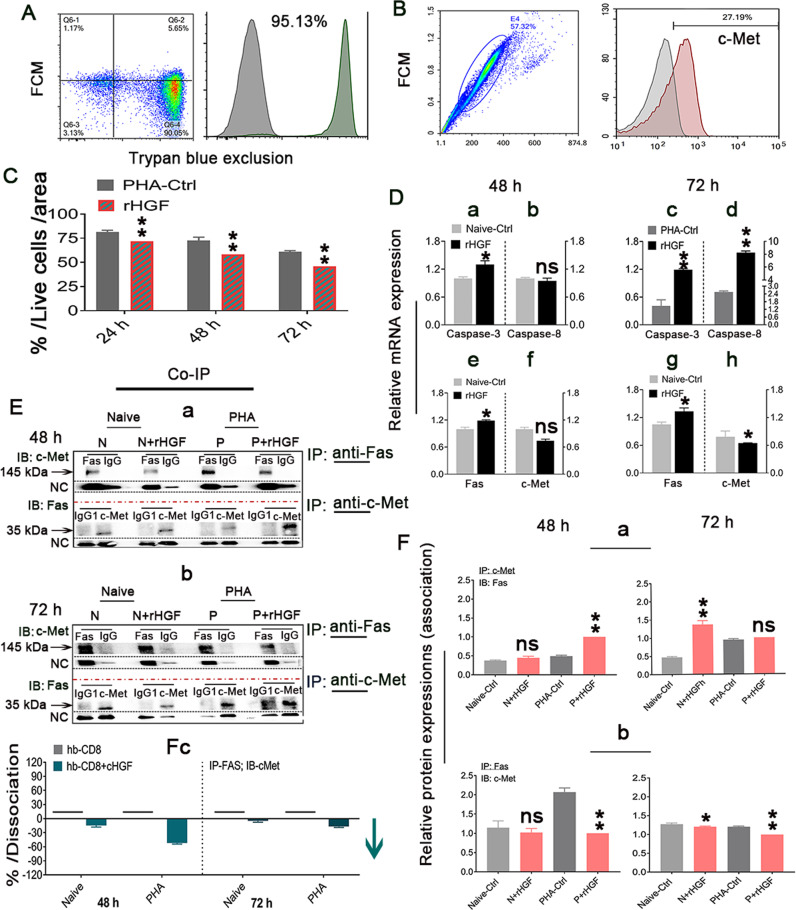
c-Met-Fas dissociation in healthy human peripheral blood CD8^+^ T cells (hp-CD8^+^ T cells) can also be induced by treatment with 400 ng/mL rHGF. **A** FCM analysis of the hpCD8^+^ CT cell purity after isolation with MACS beads (*n* = 3). **B** FCM analysis of c-Met expression in hp-CD8^+^ T cells (*n* = 3). **C** Assessment of live hp-CD8^+^ CT cells in cultures using the trypan blue exclusion assay. Cells were treated with 400 ng/mL rHGF for 24, 48, and 72 h (*n* = 3/time point). **D**(a–h) qRT‒PCR assay for assessment of the caspase-8 and caspase-3 levels in naive cells at 48 h (a, b) and PHA-stimulated hp-CD8^+^ T cells at 72 h (c, d), *n* = 6/time point; and the c-Met and Fas levels in naive hp-CD8^+^ T cells after 48 (e, f) and 72 h (g, h) of 400 ng/mL rHGF treatment, *n* = 6/time point. **E**(a, b) Co-IP assays using either IP Fas or c-Met antibody followed by IB with c-Met or Fas antibody were performed to assess the association between c-Met and Fas in naive (N) and PHA-stimulated (P) hp-CD8^+^ T cells in the presence or absence of 400 ng/mL rHGF for 48 (a, *n* = 3) and 72 h (b, *n* = 3). **F**(a–c) IP with c-Met antibody followed by IB for Fas was performed to confirm the association between c-Met and Fas in hpCD8^+^ T cells after treatment with 400 ng/mL rHGF for 48 and 72 h (a, *n* = 6); the same IP assay with Fas followed by IB with c-Met was performed with cells cocultured with 400 ng/mL rHGF for 48 and 72 h (b, *n* = 6), and the dissociation rate (a blue arrow) of c-Met from Fas was analyzed after 48–72 h of treatment with 400 ng/mL rHGF (the values of the samples marked with short horizontal lines were precisely 0) (c). The data are presented as the means ± SDs of at least three independent experiments. *p < 0.05 and **p < 0.001 indicate a statistically significant difference vs. either naive-Ctrl or PHA-Ctrl; ns represents no significance (Student’s *t* test).

We also assessed whether these findings in mice are also observed in humans. For this purpose, human hp-CD8^+^ T cells (*n* = 10) were evaluated. The hp-CD8^+^ T cells were purified by MACS and they exhibited approximately 95% purity, as determined by FCM (Fig. 6A), and approximately 27.19% of these cells expressed c-Met (Fig. 6B). As revealed by Trypan blue exclusion assays, a significantly higher proportion of live ph-CD8^+^ T cells were not viable at all tested time points during 400 ng/mL rHGF treatment (red bars) compared with that found in the non-rHGF-treated group (gray bars) (Fig. 6C). RT‒qPCR revealed that both naive and PHA-stimulated hpCD8^+^ T cells exposed to 400 ng/mL rHGF exhibited apoptotic characteristics including higher caspase-3/8 expression at 48 and 72 h compared with controls (Fig. 6Da–d), whereas increased Fas expression and decreased c-Met expression were found in naive hp-CD8^+^ T cells treated with 400 ng/mL rHGF for 48 and 72 h compared with the levels found in the control cells (Fig. 6De–h). Of note, after treatment for 48 h (Fig. 6Ea) and 72 h (Fig. 6Eb), the physical association/disassociation of the c-Met-Fas complex indicative of cell survival or apoptosis in these cells was identified by Co-IP, which suggested rHGF-specific actions. The relative protein expression levels determined by Co-IP analyses illustrated the patterns of Fas and c-Met association in both naive (Fig. 6Fb) and PHA-stimulated (Fig. 6Fa) hpCD8^+^ T cells treated for 48 and 72 h. Consistently, we also confirmed that treatment with 400 ng/mL rHGF increased the dissociation of c-Met from Fas (Fig. 6Fc), which suggested that the susceptibility of hpCD8^+^ T cells to apoptosis is dependent on the Fas/c-Met complex and thus on the HGF levels. These observations indicate that hpCD8^+^ T cells may play a role in hepatic homeostasis maintenance and potentially in the deactivation of HSCs for functional recovery by promoting fibrosis resolution during fibrosis/cirrhosis liver development. These unknown interplay signals of HGF with CD8^+^ T cells in the diseased live niche need to be further explored.

## Discussion

Studies have suggested that promoting hepatic cell repair through the prevention of apoptosis in the diseased liver by targeting death effectors, such as Fas or its downstream caspases, is a viable strategy for curtailing the progression of ESLD, such as viral hepatitis, NAFLD, alcoholic steatohepatitis, drug abuse, and autoimmune diseases [28, 29]. No effective drug for ESLD is currently available. In the present study, we describe previously unappreciated roles of HGF in inducing CD8^+^ T cell apoptosis under both physiological and pathological conditions and demonstrate that the role of HGF in apoptosis in these cells involves the dissociation of c-Met from Fas [30].

Regulation of the liver cell survival fate by targeting c-Met or its downstream effectors may also represent an effective method for curtailing progression to ESLD [31]. Some studies have shown that the c-Met peptide significantly improves liver damage by reducing liver inflammation [32, 33]. However, interestingly, our previous study using a mouse model of liver fibrosis/cirrhosis induced by DEN appears to suggest that the inflammatory environment in the fibrotic liver tissue niche is beneficial for the repair and survival of resident stem/progenitor cells by protecting them from apoptosis [5]. In the present study, we found similar evidence, which likely indicates that liver tissue-resident CD8^+^ T cells play an important role not only in infectious diseases as previously reported [34, 35] but also in improving liver damage. We found that CD8^+^ T cells in the liver fibrotic area exhibit improved survival and are associated with an improved fibrotic load compared with CD8^+^ T cells in the liver cirrhotic area by inducing α-SMA^+^ HSC apoptosis. In this context, α-SMA serves as a marker of HSC activation [36]. This finding is consistent with a recent study showing the role of CD8^+^ T cells in fibrotic resolution [26]. However, why the CD8^+^ T cells in the diseased liver niche survive during the inflammatory period but undergo apoptosis in the cirrhotic period remains unclear [5]. This phenomenon is observed regardless of whether the cells are stem/progenitor cells [5], CD8^+^ T cells (Fig. 1D, Eb) or transplanted stem/progenitor cells (not shown), and this question needs to be further investigated.

Another salient aspect of our observations is the finding that the higher levels of HGF in the LCP than in the LFP (Fig. 1A) induce CD8^+^ T cell apoptosis. we have previously shown that HGF induces apoptosis of liver-resident CD8^+^ T cells in other biological systems [21]. Similarly, Reza et al. reported [26] the beneficial of CD8^+^ T cells in liver fibrotic resolution. Correspondingly, we found in the present study of higher HGF levels in the LCP than in the LFP (Fig. 1A), where liver tissue-resident CD8^+^ T cells survive or undergo apoptosis at these respective stages. Streetz et al. study seems suggest [37] that the temporal inhibition of c-Met in recipient cells is an attractive method to improve the engraftment and selection process of transplanted hepatocytes. Our recent preclinical studies of short-term c-Met (24 h before functional cell transplantation) inhibition using anti-c-Met (HGF only receptor) antibody in DEN-induced liver fibrosis/cirrhosis before cell treatment have revealed that the cells exhibit improved functions of protecting transplant cells from apoptosis and improve functions 4 weeks after treatment (unpublished data). In the present study, because the treatment of normal CD8^+^ T cells in cultures with a higher concentration of rHGF (400 ng/mL) caused apoptosis, we hypothesize that CD8^+^ T cell death may be attributed to a cell-autonomous reaction, and such a reaction could be beneficial for immune homeostasis in the liver. Moreover, treatment with 400 ng/mL rHGF caused Fas upregulation (aggregation) (Fig. 3Bb) and c-Met downregulation (degradation) (Fig. 4A, D, E). It is therefore tempting to hypothesize that HGF disrupts the interaction between Fas and c-Met, and this effect is accompanied by DISC formation and Fas aggregation. In its aggregated form, Fas is sensitive to FasL binding, which leads to apoptosis. These results suggest that higher levels of rHGF promote the dissociation of c-Met from Fas [30]; consequently, CD8^+^ T cells undergo apoptosis. Apoptosis is believed to be a mechanism for maintaining homeostasis or improving fibrotic/cirrhotic liver development.

HGF is well known for its ability to promote hepatic cell proliferation, tissue growth and regeneration by reducing the immune response [38–40] through its only receptor, c-Met. In cultures, we indeed found that normal CD8^+^ T cell proliferation was induced by treatment with less than 5–10 ng/mL rHGF (not shown). However, when the dose of rHGF was increased to 200 ng/mL, the cells started to die via apoptosis. Such a paradoxical phenomenon is not surprising because HGF independently acts as a cytotoxic factor that was reported in the early 1990s [41]. In the present study, HGF treatment induced DISC formation and the recruitment of FADD and increased the caspase-8/3 levels in both naive and human peripheral blood normal CD8^+^ T cells. These results suggest that an association between c-Met and Fas exists in normal CD8^+^ T cells, and the present study indeed confirms this finding (Fig. 4B; Fig. 6E). Exposure to 400 ng/mL HGF disrupted the binding of c-Met to Fas. Once Fas dissociates from c-Met, the released Fas forms aggregates that exhibit enhanced sensitivity to binding to FasL, which results in the induction of apoptosis. This suggests that regulating HGF signaling may enhance the positive selection of transplanted therapeutic cells and promote functional repopulation [37]. Interestingly, the inhibition of HGF signaling by anti-c-Met-Ab prevents transplant cell apoptosis and promotes functional recovery of the cirrhotic liver (not shown), which could be clinically important. The HGF-c-Met axis elicits opposing effects on liver tissue repair by inducing niche cell survival and apoptosis. Mechanistically, it is possible that the axis downregulates HGF signals, and the binding of c-Met to Fas thus increases to prevent Fas-mediated apoptosis.

It is known that the c-Met β chain traverses the plasma membrane and harbors the tyrosine kinase and signal transduction domains, whereas the c-Met α chain connects to Fas [15]. Together, our recent discoveries using the DEN-induced liver fibrosis/cirrhosis mouse model showed that the HGFβ chain (HGFβ) is expressed at higher levels in the LFP than in the LCP. In contrast, HGF α chain (HGFα) expression exhibits the opposite trend (not shown). Thus, we hypothesize that HGFβ may be responsible for cell proliferation (survival) helping repair or regeneration through the c-Met β chain, whereas the HGF α chain may be responsible for cell apoptosis (death) making disease worse through the c-Met α chain that binds to Fas. We propose that the genes encoding the two chains may have evolved and combined to perform distinct yet complementary functions with respect to controlling the survival of liver niche cells. This mechanism would provide a novel potentially viable strategy for curtailing the progression of acute and chronic liver diseases, as mentioned above, and all of these mechanisms need to be further studied. Additionally, studies may also be warranted to establish the clinical value of the regulation of c-Met agonistic antibodies in preventing liver disease and the development of hepatocellular carcinoma (HCC) and other diseases.

In summary, the current study provides additional insights into the role of HGF signals in regulating normal and diseased liver tissue-resident CD8^+^ T cells. The study also establishes a novel paradigm through which growth factor/cytokine receptors may operate to protect cells from harmful death receptors. This finding has great implications for understanding the biological basis of hepatic niche cell regulation during physiological homeostasis and pathological disease. These findings are also of clinical significance and may provide novel information for designing future therapeutic strategies, such as strategies specifically targeting HGF α or the β, during the development of fibrosis/cirrhosis liver disease.

## Materials and methods

### Mice

Eight-to-10-week-old C57BL/6 inbred male mice weighing 22–25 g were used. All the mice were housed at the Army Medical University animal facility and maintained under specific pathogen-free conditions with a 12-h light/12-h dark cycle for two weeks before experimentation. All mouse experiments were performed following the institutional guidelines and approved by the Institutional Animal Use and Care Committee of Army Medical University (Registration number: #SYXK YU 2012–0012).

### DEN-induced liver fibrosis/cirrhosis mouse model

Liver fibrosis/cirrhosis was induced by DEN in 8–10 week, 18–22 g C57BL/6 male inbred mice [42]. DEN (0.014%, 0.13 mg/mL, 25.86 mg/kg) was supplied in the drinking water for 10 consecutive weeks, and the control mice were provided normal water until 10 weeks. Four time periods are noted in the DEN mouse model [43]: the phase of LH (0 weeks post-DEN), the liver homeostatic phase (LFP, 3–6 weeks post-DEN), the liver cirrhotic phase (LCP, 7–10 weeks post-DEN) and HCC (11–15 weeks post-DEN) [5]. In this model, approximately 10–15% mortality was observed. At least 10–15 mice were randomly assigned in all the groups were analyzed in parallel with the mice from all the other groups, and no excluded animals from analysis.

### Immunofluorescence staining

Liver tissue sections (5 µm) were fixed with 4% paraformaldehyde (PFA), submerged in 30% sucrose overnight, frozen in Tissue-Tek Optimal Cutting Temperature (OCT) compound and immunostained with primary antibodies against CD8α, Ki-67, c-Met, α-SMA and Fas (working dilution 1:400) in DMEM/F12 containing 5% normal goat serum (NGS) at 4 °C overnight for purpose target detection. A fluorescent dye (Alexa Fluor 488 or Cy3)-conjugated secondary antibody (working dilution 1:2000–4000) diluted in DMEM with 5% NGS was then added, and the sections were incubated at room temperature (RT) for 1 h and then mounted in Vectashield with 4’,6-diamidino-2-phenylindole (DAPI) to label the nuclei. The samples were rinsed again with phosphate-buffered saline (PBS) and ddH_2_O before mounting. The proportions of these stained cells in defined regions were calculated and analyzed. Three to 6 different animals were assayed with at least 16 sections/animal. Sample sizes were determined based on power estimates. All images were taken by an investigator blinded to the treatment of the individual animals. For quantification of the proportion of specific stained and unspecific stained cells was taken from multiple sections at least 2 different liver tissue regions from 3 to 6 animals.

### Isolation of CD8α-positive T cells from normal naive mouse spleen and adult peripheral blood using a MACS approach [44]

Spleens removed from mice were homogenized, and single cells were obtained in GKN solution (consisting of PBS supplemented with 11 mM D-glucose, 5.5 mM KCl, 137 mM NaCl, 25 mM Na_2_HPO_4_ and 5.5 mM NaH_2_PO_4_). The resuspended cells were then passed through a 70-mm cell strainer (Falcon) and centrifuged at 800 × *g* for 5 min. The mouse MACS method was then used to isolate the CD8α-positive cells according to the manufacturer’s guidelines. A cell purity of approximately 95% was determined by FCM with anti-mouse CD8α antibodies. The purified CD8^+^ T cells were resuspended and cocultured with different concentrations of rHGF in neurobasal A medium in the presence or absence of 1% PHA for different time periods.

hp-CD8^+^ T cells were isolated from ten healthy human peripheral blood monocytes (PBMCs). Briefly, these monocytes were then subjected to density gradient centrifugation to obtain the PBMCs. CD8^+^ T cells were isolated using a human MACS kit. The cell purity was confirmed using the above-described method, and the same cell culture conditions were used, with the exception that human-specific antibodies were used. In cultures, MACS-isolated CD8^+^ T cells were incubated for up to 72 h in 37 °C/5% CO_2_ and processed for proliferation and apoptosis analyses after treatment with 400 ng/mL rHGF. These human peripheral blood specimens were obtained from human bank of the Northwestern Hospital, Army Medical University, Chongqing China, according to a protocol approved by the University’s Institutional Review Board (Registration number: #(A)KY2022114), where collected from healthy volunteer donor peripheral blood. Informed consent was obtained from all subjects.

### Assessment of apoptosis by TUNEL assay

A One-Step TUNEL Apoptosis Assay Kit was used for the assessment of liver tissue-resident CD8+ and α-SMA^+^ HSCs in accordance with the manufacturer’s instructions. Briefly, sections of paraffin-embedded hepatic tissues were deparaffinized in toluene and then dehydrated in ethanol solutions. After three 3-min washes with PBS, the sections were incubated with 10 g/mL DNase I at room temperature for 10 min to cause DNA nicking. Negative control sections were not subjected to TUNEL enzyme treatment. The sections were blocked with 5% bovine serum albumin (BSA) for 20 min and incubated with 50 μL of TUNEL at 37 °C in a humid chamber for 60 min. The samples were washed again with Tris-buffered saline (TBS), and DAPI was added for the detection of apoptotic cells based on blue‒black nuclear staining. One hundred random fields under a fluorescence microscope (original magnification: ×200) were counted by a researcher in a blinded manner. Images were obtained using an Olympus BX53 microscope.

### CCK-8 assay

The CD8^+^ T cells were cultured in an incubator at 37 °C with 5% CO_2_. 2-(2-methoxy-4-nitrophenyl)-3-(4-nitrophenyl)-5-(2,4-sulfophenyl)-2*H*tetrazolemonosodium salt (WST-8) [45] was added to the plate (10 μL/well, once every 24 h) for 3 days. A Varioskan Flash Spectral Scanning Multimode microplate reader (Thermo Scientific, MA, USA) was used to detect the optical density (OD) at 450 nm, and the cytokinetics of the cells were analyzed as described by SkanIt RE using Varioskan Flash 2.4.3.

### Co-IP and IB [46]

Cell lysates were immunoprecipitated with an antibody against either Fas (IP Fas) or c-Met (IP c-Met) and then subjected to IB with either anti-c-Met antibodies to detect c-Met (IB for c-Met) or anti-Fas antibodies to detect Fas (IB for Fas) and determine the binding of c-Met to Fas. In addition, IB with antibodies against FADD, caspase-8 and caspase-3 was also performed to assess the formation of DISC and to confirm the Fas mediated apoptosis in CD8^+^ T cells (2–4 × 10^7^/well). Jurkat T cells (denoted J) express Fas [47] but not c-Met [48]. The Jurkat T cells were gifted from Professor Lui of our University who purchased the cells from ATCC (Clone E6-1, cat no. TIB152). we used the cell line as positive controls. Briefly, an equal amount of cell lysate (500 µg to 1 mg) was incubated with protein A/G magnetic beads in IP lysis buffer at RT for 2 h and subjected to three 3-min washes with 0.1% Tween 20 in TBS. Protein A/G magnetic beads were then incubated with a mouse IgG1 isotype control antibody (2 µg/mL), a rabbit IgG isotype control antibody (2 µg/mL) or a mouse anti-c-Met monoclonal or rabbit polyclonal anti-Fas antibody (2 µg/mL) for 1 h at RT. The protein A/G magnetic beads were centrifuged at 10,000 × *g* and washed thrice with lysis buffer. The pellet was resuspended in Western blot loading buffer and heated at 95 °C for 5 min. After centrifugation, the supernatant was resolved on 6% and 12.5% sodium dodecyl sulfate‒polyacrylamide gel electrophoresis (SDS‒PAGE) gels for Western blot analysis. For the examination of protein–protein interactions, co-IP with both c-Met and Fas antibodies was performed. A Western blot analysis was then performed using an antibody against the associated protein (Fas or c-Met). Total cell lysates (2–4 × 10^7^ untreated or treated cells) were generated in DISC lysate buffer and resolved on 12.5% SDS‒PAGE gels. IB was then performed to evaluate the expression of FADD, caspase-8 and caspase-3.

### Western blot assay [49]

Total proteins were extracted from liver tissues, and specific proteins were detected by Western blotting. The protein concentration of liver lysates was estimated using a biochemical analysis of proteins (bicinchoninic acid [BCA] protein assay kit) according to the manufacturer’s protocol. The lysates (20 μg/lane) were subjected to heat denaturation, separated on 10% SDS–PAGE gels, and subsequently electrotransferred onto a polyvinylidene difluoride membrane. The membranes were blocked with 5% BSA for 90 min at RT and incubated with the corresponding primary antibodies against HGF, caspase-8 (1:500) and glyceraldehyde-3-phosphate dehydrogenase (GAPDH) (control, 1:500) in blocking solution at 4 °C overnight. After several 7-min washes with TBST, the protein signals were determined with secondary horseradish peroxidase (HRP)-conjugated antibody at RT for 90 min. The target proteins were then visualized using an enhanced chemiluminescence system (ECL, Pierce, Bonn, Germany).

### RT–qPCR assay [50]

RNA extraction was performed using a HiPure Total RNA Plus Mini Kit. Eighty nanograms of total RNA was transcribed into cDNA using the Prime Script RT Reagent Kit. The cDNA expression of the *hgf* gene was quantified with TB Green-Premix Ex Tag (Takara) and assessed using a quantitative PCR system (CFX96 Real-Time System, Bio-Rad, Hercules, CA, USA). Here, *gapdh* served as a housekeeping gene for normalization. Relative gene expression was determined using the 2^−ΔΔCt^ method. The following *hgf* primer set was used: forward, 5’-ACT TCT GCC GGT CCT GTT G-3’; reverse, 5’-CCC CTG TTC CTG ATA CAC CT-3’.

All information on the primary antibodies, secondary antibodies, reagents and their related measurement kits are listed in Table 1.

**Table 1:** Antibodies (Abs)/reagents/kits.

Items	Abs	Cat no.	Applications	Company	City or State	Country
Primary Abs	CD8α	14-0081-85	WB, IHC, IF, FC, IP, Neu, FN	Invitrogen	California	USA
	CD8	Sc-1177	WB, IP, IF, FC	Santa Cruz Biotechnology	California	USA
	Ki67	550609	FC, IF	Biosciences	New Jersey	USA
	c-Met	bs-0668R	ELISA, IHC, IHC-FFC, IF	Bioss	Beijing	China
		3127s	WB, IP	CST	Danvers, Massachusetts	USA
	α-SMA	Sc-130617	WB, IP, IF, IHC(P), ELISA	Santa Cruz Biotechnology	California	USA
	Fas	Sc-74540	WB, IP, IF ELISA			
Secondary Abs	Alexa Fluor 488	115-545-003	IF	Jackson	Philadelphia	USA
	Cy3	115-165-003	IF			
Reagents	DEN	N0756		Sigma‒Aldrich	Darmstadt	Germany
	Sucrose	V900116				
	OCT	4583		SAKURA	California	USA
	NGS	AR0009	Blockage	BOSTER	California	USA
	DAPI	C1006	Apoptosis Assays	Beyotime	Shanghai	China
	PHA					
	Gapdh	60004-1-Lg	WB, IP, IF, FC	Proteintech	Wuhan	China
Kits	TUNEL	C1090	Apoptosis Assays	Beyotime	Shanghai	China
	BCA	P0010				
	CCK-8	c0043				
	MACS	(human) 557941		Biosciences	New Jersey	USA
		(mouse) 558471				

### Statistical analyses

All summary data are presented as the means ± standard deviations (SDs). Two-group comparisons were performed using Student’s *t* tests. Multiple group comparisons were performed by one‐way analysis of variance (ANOVA) followed by Bonferroni’s post hoc test. All statistical analyses were performed using SPSS software (version 13.0, SPSS Inc., Chicago, IL, USA). *P* < 0.05 (two-sided) was considered to indicate statistical significance. At least three independent experiments were performed and the data are estimated as the means ± SDs. The data meet the assumptions of the tests.

## Data Availability

All data generated or analyzed during this study are included in this published article and its Supplementary Information files.

## References

[1] Canbay A, Friedman S, Gores GJ (2004). Apoptosis: the nexus of liver injury and fibrosis. Hepatology..

[2] Feldstein AE, Canbay A, Angulo P, Taniai M, Burgart LJ, Lindor KD (2003). Hepatocyte apoptosis and Fas expression are prominent features of human nonalcoholic steatohepatitis. Gastroenterology..

[3] Rodina AS, Shubina ME, Kurbatova IV, Topchieva LV, Dudanova OP (2022). Hepatocytic apoptosis and immune dysfunction in decompensation of alcoholic liver cirrhosis with different grades of acute-on-chronic liver failure. Bull Exp Biol Med.

[4] Horn CL, Morales AL, Savard C, Farrell GC, Ioannou GN (2022). Role of cholesterol-associated steatohepatitis in the development of NASH. Hepatol Commun.

[5] Chen Q, You X, Yang W, Jiang S, Lai J, Zhang H (2020). Survival of endogenous hepatic stem/progenitor cells in liver tissues during liver cirrhosis. Life Sci.

[6] Koyama Y, Brenner DA (2017). Liver inflammation and fibrosis. J Clin Invest.

[7] Cheng ML, Nakib D, Perciani CT, MacParland SA (2021). The immune niche of the liver. Clin Sci..

[8] Hayakawa Y, Nakagawa H, Rustgi AK, Que J, Wang TC (2021). Stem cells and origins of cancer in the upper gastrointestinal tract. Cell Stem Cell.

[9] Premkumar M, Anand AC (2022). Overview of complications in cirrhosis. J Clin Exp Hepatol.

[10] Feldstein AE, Canbay A, Guicciardi ME, Higuchi H, Bronk SF, Gores GJ (2003). Diet associated hepatic steatosis sensitizes to Fas mediated liver injury in mice. J Hepatol.

[11] Engin A (2017). Non-alcoholic fatty liver disease. Adv Exp Med Biol.

[12] Stoffers P, Guckenbiehl S, Welker MW, Zeuzem S, Lange CM, Trebicka J (2022). Diagnostic and prognostic significance of cell death markers in patients with cirrhosis and acute decompensation. PLoS ONE.

[13] Bhandari K, Kapoor D (2022). Fatigue in cirrhosis. J Clin Exp Hepatol.

[14] Bottaro DP, Rubin JS, Faletto DL, Chan AM, Kmiecik TE, Vande Woude GF (1991). Identification of the hepatocyte growth factor receptor as the c-met protooncogene product. Science..

[15] Wang X, DeFrances MC, Dai Y, Pediaditakis P, Johnson C, Bell A (2002). A mechanism of cell survival: sequestration of Fas by the HGF receptor Met. Mol Cell..

[16] Huh CG, Factor VM, Sánchez A, Uchida K, Conner EA, Thorgeirsson SS (2004). Hepatocyte growth factor/c-met signaling pathway is required for efficient liver regeneration and repair. Proc Natl Acad Sci USA.

[17] Birchmeier C, Birchmeier W, Gherardi E, Woude GF (2003). Met, metastasis, motility and more. Nat Rev Mol Cell Biol..

[18] Trusolino L, Comoglio PM (2002). Scatter-factor and semaphorin receptors: cell signalling for invasive growth. Nat Rev Cancer..

[19] Zou C, Ma J, Wang X, Guo L, Zhu Z, Stoops J (2007). Lack of Fas antagonism by Met in human fatty liver disease. Nat Med.

[20] Gohda E, Takebe T, Sotani T, Nakamura S, Minowada J, Yamamoto I (1998). Induction of hepatocyte growth factor/scatter factor by interferon-gamma in human leukemia cells. J Cell Physiol.

[21] Chen Q, You Y, Zhang Y, Zhang H, Bai L (2021). Hepatocyte growth factor mediates a novel form of hepatic stem/progenitor cell-induced tolerance in a rat xenogeneic liver rejection model. Int Immunopharmacol.

[22] Nagata S (1996). Fas-mediated apoptosis. Adv Exp Med Biol.

[23] Ashkenazi A, Dixit VM (1998). Death receptors: signaling and modulation. Science..

[24] Bremner TA, Chatterjee D, Han Z, Tsan MF, Wyche JH (1999). THP-1 monocytic leukemia cells express Fas ligand constitutively and kill Fas-positive Jurkat cells. Leuk Res.

[25] Efferth T, Fabry U, Osieka R (1996). Anti-Fas/Apo-1 monoclonal antibody CH-11 depletes glutathione and kills multidrug-resistant human leukemic cells. Blood Cells Mol Dis.

[26] Koda Y, Teratani T, Chu PS, Hagihara Y, Mikami Y, Harada Y (2021). CD8 + tissue-resident memory T cells promote liver fibrosis resolution by inducing apoptosis of hepatic stellate cells. Nat Commun.

[27] Liu X, Mi X, Wang Z, Zhang M, Hou J, Jiang S (2020). Ginsenoside Rg3 promotes regression from hepatic fibrosis through reducing inflammation-mediated autophagy signaling pathway. Cell Death Dis.

[28] Feldstein A, Gores GJ (2004). Steatohepatitis and apoptosis: therapeutic implications. Am J Gastroenterol..

[29] Guicciardi ME, Gores GJ (2004). Cheating death in the liver. Nat Med..

[30] Smyth LA, Brady HJ (2005). cMet and Fas receptor interaction inhibits death-inducing signaling complex formation in endothelial cells. Hypertension..

[31] Zhao Y, Ye W, Wang YD, Chen WD (2022). HGF/c-Met: a key promoter in liver regeneration. Front Pharmacol.

[32] Tang LS, Covert E, Wilson E, Kottilil S (2018). Chronic hepatitis B infection: a review. JAMA..

[33] Kalas MA, Chavez L, Leon M, Taweesedt PT, Surani S (2021). Abnormal liver enzymes: a review for clinicians. World J Hepatol.

[34] Fernandez-Ruiz D, Ng WY, Holz LE, Ma JZ, Zaid A, Wong YC (2016). Liver-resident memory CD8 + T cells form a front-line defense against malaria liver-stage infection. Immunity..

[35] Wakim LM, Waithman J, Rooijen N, Heat WR, Carbone FR (2008). Dendritic cell-induced memory T cell activation in nonlymphoid tissues. Science..

[36] Li Y, Fan W, Link F, Wang S, Dooley S (2021). Transforming growth factor β latency: a mechanism of cytokine storage and signalling regulation in liver homeostasis and disease. JHEP Rep.

[37] Kaldenbach M, Giebeler A, Tschaharganeh DF, Erschfeld S, Wasmuth HE, Dolle L (2012). Hepatocyte growth factor/c-Met signalling is important for the selection of transplanted hepatocytes. Gut..

[38] Bai L, Lennon DP, Caplan AI, DeChant A, Hecker J, Kranso J (2012). Hepatocyte growth factor mediates mesenchymal stem cell–induced recovery in multiple sclerosis models. Nat Neurosci.

[39] Michalopoulos GK, DeFrances MC (1997). Liver regeneration. Science..

[40] Amicone L, Spagnoli FM, Späth G, Giordano S, Tommasini C, Bernardini S (1997). Transgenic expression in the liver of truncated Met blocks apoptosis and permits immortalization of hepatocytes. EMBO J.

[41] Shima N, Itagaki Y, Nagao M, Yasuda H, Morinaga T, Higashio K (1991). A fibroblast-derived tumor cytotoxic factor/F-TCF (hepatocyte growth factor/HGF) has multiple functions in vitro. Cell Biol Int Rep.

[42] Tolba R, Kraus T, Liedtke C, Schwarz M, Weiskirchen R (2015). Diethylnitrosamine (DEN)-induced carcinogenic liver injury in mice. Lab Anim.

[43] Zhang H, Siegel CT, Shuai L, Lai J, Zeng L, Yujun Z (2016). Repair of liver mediated by adult mouse liver neuro-glia antigen 2-positive progenitor cell transplantation in a mouse model of cirrhosis. Sci Rep.

[44] Hübner A, Derkow K, Bräuer AU (2014). Efficient isolation of CD8α positive T cells from postnatal mice using a combined MACS approach. J Immunol Methods.

[45] Wu X, Fu J, Mei R, Dai X, Li J, Zhao X (2022). Inhibition of liver cancer HepG2 cell proliferation by enzymatically prepared low-molecular citrus pectin. Curr Pharm Biotechnol.

[46] Takahashi Y (2015). Co-immunoprecipitation from transfected cells. Methods Mol Biol.

[47] Connell JO, Sullivan GC, Collins JK, Shanahan F (1996). The Fas counterattack: Fas-mediated T cell killing by colon cancer cells expressing Fas ligand. J Exp Med..

[48] Abraham RT, Weiss A (2004). Jurkat T cells and development of the T-cell receptor signalling paradigm. Nat Rev Immunol..

[49] Shabir S, Kaul B, Pachnio A, Banham GD, Smith H, Chand S (2013). Impaired direct priming of CD8 T cells by donor-derived cytomegalovirus following kidney transplantation. J Am Soc Nephrol..

[50] Chan KK, Hon TC, Au KY, Choi HL, Wong DK, Chan AC (2022). Stanniocalcin 1 is a serum biomarker and potential therapeutic target for HBV-associated liver fibrosis. J Pathol.

